# Cache-Aided General Linear Function Retrieval

**DOI:** 10.3390/e23010025

**Published:** 2020-12-26

**Authors:** Kai Wan, Hua Sun, Mingyue Ji, Daniela Tuninetti, Giuseppe Caire

**Affiliations:** 1 Electrical Engineering and Computer Science Department, Technische Universität Berlin, 10587 Berlin, Germany; caire@tu-berlin.de; 2 Department of Electrical Engineering, University of North Texas, Denton, TX 76203, USA; hua.sun@unt.edu; 3 Electrical and Computer Engineering Department, University of Utah, Salt Lake City, UT 84112, USA; mingyue.ji@utah.edu; 4 Electrical and Computer Engineering Department, University of Illinois Chicago, Chicago, IL 60607, USA; danielat@uic.edu

**Keywords:** coded caching, linear function retrieval, uncoded cache placement

## Abstract

Coded Caching, proposed by Maddah-Ali and Niesen (MAN), has the potential to reduce network traffic by pre-storing content in the users’ local memories when the network is underutilized and transmitting coded multicast messages that simultaneously benefit many users at once during peak-hour times. This paper considers the linear function retrieval version of the original coded caching setting, where users are interested in retrieving a number of linear combinations of the data points stored at the server, as opposed to a single file. This extends the scope of the authors’ past work that only considered the class of linear functions that operate element-wise over the files. On observing that the existing cache-aided scalar linear function retrieval scheme does not work in the proposed setting, this paper designs a novel coded caching scheme that outperforms uncoded caching schemes that either use unicast transmissions or let each user recover all files in the library.

## 1. Introduction

Content caching is an efficient technique to handle the increase of requests for massive amounts of data and content over communication networks. By leveraging low-cost memory components at the user sides, caching reduces peak-time traffic by prefetching contents closer to users during off-peak time, thereby reducing the transmission delay or equivalently increasing the bandwidth in communication systems. Traditional caching techniques aim at prefetching popular content by predicting the user demands, thus realizing a “local caching gain” (i.e., that scales with the amount of local memory) [1]. Maddah-Ali and Niesen (MAN) showed that it is possible to actually attain a “global caching gain” (i.e., that scales with the global amount of memory in the network) by using codes [2]. The idea is that, if a single transmission can serve a number of users simultaneously, the network load can be reduced by the same factor thus speeding-up communications significantly.

In the MAN setting, a server has a library of N files and broadcasts to K users through an error-free shared-link. Each user has a cache of size of at most M files. The MAN scheme consists of two phases: placement phase, where the server pushes content from the library to the local caches without knowledge of user future demands, and delivery phase, where each user requests one file and the server broadcasts coded packets such that each user can correctly recover its desired file. The objective is to minimize the worst-case load over all possible user demands, that is, the number of files that must be communicated so that any demands can be satisfied. The MAN scheme is optimal under the constraint of uncoded cache placement (i.e., each user directly stores a collection of segments of the library files in its cache) when N≥K [3,4]. By removing the redundant transmissions in the MAN scheme when a file is requested multiple times, Yu, Maddah-Ali, and Avestimehr (YMA) derived a scheme that is optimal under the constraint of uncoded cache placement for N<K [5]. In general, the YMA scheme is order optimal to within a factor of 2 [6], that is, coded placement can at best half the load of the YMA scheme.

On the motivation that linear and multivariate polynomial queries naturally arise in modern engineering problems and deep learning algorithms such as matrix-vector, matrix-matrix multiplications, in [7] the authors posed the question of what is the optimal worst-case load when the cache-aided users are interested in retrieving a scalar linear function of the files rather than a single file. For the class of functions considered in [7], which are restricted to operate element-wise on the file entries, it was surprisingly shown that the YMA load can be achieved, that is, there is no penalty in terms of load in retrieving scalar linear functions under the constraint of uncoded cache placement. It was noted in [7] that the proposed scalar linear function scheme can be extended to all scenarios to which the original MAN scheme has been extended, such as for example demand-private retrieval [8] and Device-to-Device networks [9,10]. In addition, the scalar linear function scheme [7] can be used as a building block to provide demand-privacy and content-security against colluding users [11,12].

In this paper, we move to a more general case of cache-aided linear function retrieval than in [7], where users can request general linear combinations of all symbols in the library, and not necessarily restricted to operate element-wise on the file entries. For example, each user aims to compute some statistics of a bunch of data such as local weighted averages (which are general linear functions) of the data; these are very common tasks in many applications depending on the data and on the weights.

Instead, each user may want to compute some statistics of a bunch of data such as average, or compute local weighted averages (which are general linear functions) of the data. We think that it is a very common task in many applications depending on the data and on the weights. So in our paper, if the Academic Editor agrees, we will replace the application in deep neutral networks by the application in computing local weighted averages.

Besides the novel and realistic problem formulation, our main contributions are as follows. We first introduce a baseline scheme that either lets each user recover all the symbols in the library or uses unicast transmissions to satisfy each user. The main challenge to implement a coded caching strategy in this problem is that each symbol in a user’s demand is a linear combination of all the symbols in the library. Inspired by the grouping coded caching strategy in [13], which was used to reduce the sub-packetization level (The sub-packetization level is the smallest file length necessary to realize an achievable scheme.), we propose a scheme that treats the demand of each user as a matrix-vector multiplication and uses the grouping strategy to generate multicast messages after possibly performing invertible linear matrix operations. The proposed scheme outperforms the baseline scheme in all parameter regimes.

### 1.1. Paper Organization

The rest of this paper is organized as follows. Section 2 formulates the shared-link cache-aided general linear function retrieval problem. Section 3 provides the main result of this paper. Section 4 provides some numerical evaluations. Section 5 concludes the paper. Some proofs may be found in Appendices.

### 1.2. Notation Convention

Calligraphic symbols denote sets, bold symbols denote vectors and matrices, and sans-serif symbols denote system parameters. We use |·| to represent the cardinality of a set or the length of a vector; $\left[a:b\right]:=\left\{a,a+1,\ldots ,b\right\}$ and [n]:=[1:n]; ⊕ represents bit-wise XOR; ${\left[a\right]}^{+}:=\max\left\{a,0\right\}$; $F_{q}$ represents a finite field with order q; $A^{T}$ and $A^{-1}$ represent the transpose and the inverse of matrix A, respectively; ${\mathrm{rank}}_{q}\left(A\right)$ represents the rank of matrix A on field $F_{q}$; $I_{n}$ represents the identity matrix with dimension n×n; ${\left(A\right)}_{m\times n}$ represents the dimension of matrix A is m×n; we let xy=0 if x<0 or y<0 or x<y.

## 2. System Model

Different from [7], here we consider the case where the users’ desired linear functions are no longer scalar or operating element-wise across the files entries, thus we consider the whole library as a single file.

The (K,F,L,q) shared-link cache-aided general linear function retrieval problem consists of a central server with access to a library of F independent and identically distributed (i.i.d.) symbols over a finite filed $F_{q}$, denoted by $w={\left(w_{1},\ldots ,w_{F}\right)}^{T}\in {\left(F_{q}\right)}^{F}.$ We often treat w as a column vector, which should be clear from the context. The server is connected to K cache-aided users through an error-free shared-link. The system has two phases.

- In the **placement phase**, the server pushes up to M symbols into the local cache of each user, where M∈[0:F], without knowing what the users will demand later. The cached content of user k∈[K] is denoted by
  (1) $$\begin{array}{c} Z_{k}=\phi_{k}\left(w\right), \end{array}$$
  where $\phi_{k}$ is the placement function for user *k* defined as
  (2) $$\begin{array}{cc} \phi_{k} & :{\left(F_{q}\right)}^{F}\to {\left(F_{q}\right)}^{M},\;k\in \left[K\right]. \end{array}$$
  M is referred to as the cache (or memory) size. If each user directly copies M symbols from the library into its cache, the cache placement is said to be uncoded.
- In the **delivery phase**, each user wants to retrieve L linear combinations of all the symbols in the library, where L∈[1:F].
  The demand of user k∈[K] is represented by the matrix $D_{k}\in {\left(F_{q}\right)}^{L\times F}$, meaning user *k* aims to retrieve
  (3) $$\begin{array}{c} y_{k}=D_{k}\;w\in {\left(F_{q}\right)}^{L}, \end{array}$$
  Let the collection of all demand matrices be $D:=\left[D_{1};\ldots ;D_{K}\right]\in {\left(F_{q}\right)}^{KL\times F}$. We assume that the server and all users know D which is communicated on a separate channel, thus not impacting the downlink load next—see also Remark 4. ( Notice that differently from the cache-aided matrix multiplication problem in [14], where the matrix on the each side of the desired multiplication is one of the library files, in this paper each user k∈[K] desires $D_{k}w$ where $D_{k}$ is known by all the users in the delivery phase and w represents the vector of all symbols in the library.)
  According to all the users’ demand matrix D, the server broadcasts the message
  (4) $$\begin{array}{c} X=\psi (D,w), \end{array}$$
  where ψ is the encoding function
  (5) $$\begin{array}{cc} \psi & :{\left(F_{q}\right)}^{KL\times F}\times {\left(F_{q}\right)}^{F}\to {\left(F_{q}\right)}^{R}, \end{array}$$
  for some R∈[0:F]. R is referred to as the load.

*Achievability:* For the (K,F,L,q) shared-link cache-aided general linear function retrieval problem, we say that the pair (M,R) is achievable if for any possible demand D there exist placement functions in (2) and a delivery function in (5) such that

(6) $$\begin{array}{c} H\left(D_{k}w|D,Z_{k},X\right)=0,\;\forall k\in \left[K\right]. \end{array}$$

*Optimal memory-load tradeoff:* For the (K,F,L,q) shared-link cache-aided general linear function retrieval problem, the objective is to determine the minimum worst-case downlink load (or load for simplicity) defined as

(7) $$\begin{array}{c} R^{★}\left(M\right)=\min_{\phi_{1},\ldots ,\phi_{K},\psi }\left\{R:\left(M,R\right)\;\mathrm{is}\;\mathrm{achievable}\right\}. \end{array}$$

*Optimal memory-load tradeoff in the limit for large file size:* Since solving the problem in (7) for any given (K,F,L,q) is challenging, in the following we shall consider the regime where the file size F is as large as desired and we thus let the system parameters scale with the file length as follows

(8) $$\begin{array}{cc} M & :=\mu F,\;\mu \in [0,1], \end{array}$$

(9) $$\begin{array}{cc} L & :=\lambda F,\;\lambda \in [0,1], \end{array}$$

(10) $$\begin{array}{cc} R & :=\rho F,\;\rho \in [0,1]. \end{array}$$

For fixed (K,λ) we aim to characterize the minimum worst-case normalized downlink load (or normalized load for simplicity)

(11) $$\begin{array}{c} \rho^{★}\left(\mu \right)=\min_{\phi_{1},\ldots ,\phi_{K},\psi }\left\{\rho :\left(M,R)=(\mu F,\rho F\right)\;\mathrm{is}\;\mathrm{achievable}\;\mathrm{for}\;\mathrm{some}\;\left(F,q\right)\right\}. \end{array}$$

**Remark** **1** (Relationship to [7])**.** *The cache-aided scalar linear function retrieval problem in [7] is a special case of the formulation here. More precisely, let F=NL (i.e., $\frac{1}{N}=\lambda$), where N indicates the number of files and λF is the file length. The demand of user k∈[K] is represented by the vector $y_{k}=\left(y_{k,1},y_{k,2},\ldots ,y_{k,N}\right)\in {\left(F_{q}\right)}^{N}$ by which we mean that the user is requesting*

(12) $$\begin{array}{c} D_{k}=\left[\begin{array}{c} y_{k,1}I_{L},\;y_{k,2}I_{L},\ldots ,y_{k,N}I_{L} \end{array}\right]\in {\left(F_{q}\right)}^{L\times NL}, \end{array}$$

*where $I_{n}$ is the identity matrix with dimension n×n. In the restricted setting where the demands are as in (12) the optimal load under the constraint of uncoded cache placement is the lower convex envelop of the points*

(13) ML,RscalarL=NtK,Kt+1−K−min{K,N}t+1Kt,t∈[0:K],

(14) ⟺(μ,ρscalar)=tK,λKt+1−K−min{K,N}t+1Kt,t∈[0:K],

*where for a given value of t in (13) the subpacketization level L must be an integer multiple of Kt.*

**Remark** **2** (A minrank solution)**.** *For the (K,F,L,q) shared-link cache-aided general linear function retrieval problem, the best linear scheme, inspired by [15,16], is a follows. Linear placement: user k∈[K] caches $Z_{k}=P_{k}w\in {\left(F_{q}\right)}^{M}$ for some $P_{k}\in {\left(F_{q}\right)}^{M\times F}$. Linear delivery: the server sends, in the worst case, a number of symbols given by*

(15) $$\begin{array}{c} R_{\mathrm{minrank}}=\min_{P_{1},\ldots ,P_{K}}\max_{D_{1},\ldots ,D_{K}}\min_{T_{1},\ldots ,T_{K}}\mathrm{rank}\left[\begin{array}{c} D_{1}+T_{1}P_{1}\\ D_{2}+T_{2}P_{2}\\ \vdots \\ D_{K}+T_{K}P_{K} \end{array}\right], \end{array}$$

*where $T_{k}\in {\left(F_{q}\right)}^{L\times M},\;k\in \left[K\right]$.*

*Solving the minrank problem in (15) is hard [15,16], thus in the following we shall design a scheme with lower complexity.*

**Remark** **3** (A baseline scheme)**.** *For the (K,F,L,q) shared-link cache-aided general linear function retrieval problem, the load*

(16) $$\begin{array}{cc} R_{\mathrm{baseline}} & =\min\left\{KL,F-M\right\}\\ ⟺\;\rho_{\mathrm{baseline}} & =\min\left\{K\lambda ,1-\mu \right\}, \end{array}$$

*can be achieved by an uncoded caching strategy as follows.*

- In order to achieve the load KL, we transmit one by one the elements of $y_{k},k\in \left[K\right],$ in (3). The main limitation of this unicast transmission scheme is the lack of multicast gain.
- In order to achieve F−M we let each user recover all the symbols in the library. In the placement phase, each user caches the first M symbols in the library. In the delivery phase, the server transmits all the remaining F−M symbols. The main limitation of this scheme is that, if L<F−M, the users do not need to recover all the symbols in the library in order to retrieve their desired function.

*The main contribution of this paper is to find schemes that, despite the lack of structure on the demand matrices in general, achieve a smaller load than (16).*

**Remark** **4** (Uplink and downlink loads)**.** *Besides downlink load, uplink load is also considered in the distributed matrix-matrix multiplication problem [17,18,19]. In this work, the communication cost of uploading the demand matrix to the server is not a focus, i.e, we assume that each user communicates the whole demand matrix to the server and all other users on a separate channel that is not the bottleneck in the system. This assumption can be also justified as follows. Let D(k) denotes the set of possible demand matrices of user k∈[K], referred to as demand range, that is, user k chooses one matrix in D(k) as its demand. We assume that D(k) is known by the server and all users. The communication cost to let the server and the other users know the realization of the demand matrix is negligible compared to the number of transmissions from the server if $\sum_{k\in [K]}\log_{q}\left(|D\left(k\right)|\right)\ll F$.*

## 3. Main Result

Based on Remark 3, the main challenge is to design a coded caching strategy that (i) lets each user directly recover the desired linear combinations, instead of recovering all the library symbols, and (ii) attains coded caching gain, as opposed to serving the users one-by-one with unicast transmissions. The main contribution of this paper is the following theorem, which is proved in Appendix A.

**Theorem** **1.** *For the (K,λ) shared-link cache-aided general linear function retrieval problem, we have:*

- *if $\mu =\alpha \frac{g-1}{g}+\left(1-\alpha \right)\frac{g}{g+1}$ where g∈[K−1] and α∈[0,1], the following normalized load is achievable*
  (17) $$\begin{array}{cc} \rho_{\mathrm{ach}} & :=\left\{\begin{array}{cc} \left\lceil \frac{K}{g}\right\rceil \lambda , & \;\;\mathrm{if}\;\frac{\alpha }{g}\geq \left\lceil \frac{K}{g}\right\rceil \lambda \\ \; & \\ \min\left\{\rho_{1},\rho_{2}\right\}\; & \;\;\mathrm{if}\;\frac{\alpha }{g}\leq \left\lceil \frac{K}{g}\right\rceil \lambda \end{array}\right., \end{array}$$
  (18) $$\begin{array}{cc} \rho_{1} & :=\frac{\alpha }{g}+\min\left\{\left\lceil \frac{K}{g+1}\right\rceil \lambda ,\frac{\left(1-\alpha \right)}{g+1}\right\}, \end{array}$$
  (19) $$\begin{array}{cc} \rho_{2} & :=\left\lceil \frac{K}{g}\right\rceil \min\left\{\frac{\alpha }{g},\lambda \right\}+\min\left\{\left\lceil \frac{K}{g+1}\right\rceil {\left[\lambda -\frac{\alpha }{g}\right]}^{+},\frac{\left(1-\alpha \right)}{g+1}\right\}; \end{array}$$
- *if $\mu =\alpha \frac{K-1}{K}+\left(1-\alpha \right)$ where α∈[0,1], the following normalized load is achievable*
  (20) $$\begin{array}{c} \rho_{\mathrm{ach}}=\rho_{3}=\min\left\{\frac{\alpha }{K},\lambda \right\}. \end{array}$$

Next, we provide the intuition behind the proposed scheme in Theorem 1, which is based on three ingredients:

1. We start by the achievable scheme for (20) with α=1. We aim to design the cache placement such that each user caches a fraction $\frac{K-1}{K}$ of the file and the uncached part of file by this user is known by the remaining K−1 users. With this cache placement, the delivery consists of a single multicast message with multicasting gain K. More precisely, the construction of the proposed scheme is as follows.
  ecalling that, in Remark 1 with t=K−1, each user misses a fraction 1/K of each file and that missing fraction is known by the remaining K−1 users; with t+1=K, the delivery consists of a single multicast message with multicasting gain K that is the sum of each user’s missing fraction of the demanded file. In our context, this idea becomes the following scheme.
  Assume K divides F. We use here a Matlab-like notation for submatrices. The library is partitioned into K equal length subfiles as follows
  (21) $$\begin{array}{cc} I_{k} & :=\left[\left(k-1\right)\frac{F}{K}+1:k\frac{F}{K}\right],k\in \left[K\right], \end{array}$$
  (22) $$\begin{array}{cc} w_{k} & :=w\left(I_{k}\right)\in {\left(F_{q}\right)}^{\frac{F}{K}},k\in \left[K\right], \end{array}$$
  (23) $$\begin{array}{cc} w & =(w_{1},\ldots ,w_{K}); \end{array}$$
  user k∈[K] caches $Z_{k}=\left(w_{j}:j\in \left[K\right]\backslash \left\{k\right\}\right)$; the server delivers the multicast message
  (24) $$\begin{array}{c} X=\left\{\begin{array}{cc} \sum_{k\in [K]}D_{k;\;:,I_{k}}w_{k}\in {\left(F_{q}\right)}^{L}, & \mathrm{if}\;\frac{F}{K}>L\\ \sum_{k\in [K]}w_{k}\in {\left(F_{q}\right)}^{\frac{F}{K}}, & \mathrm{if}\;\frac{F}{K}\leq L \end{array}\right., \end{array}$$
  where $D_{k;\;:,I_{k}}$ represents the sub-matrix of $D_{k}$ including the columns with indices in $I_{k}$. In *X*, each user k∈[K] knows all but the requested vector
  $$\begin{array}{c} \left\{\begin{array}{cc} D_{k;\;:,I_{k}}w_{k}, & \mathrm{if}\;\frac{F}{K}>L;\\ w_{k}, & \mathrm{if}\;\frac{F}{K}\leq L, \end{array}\right., \end{array}$$
  such that user *k* can recover either of them. Thus an achieved normalized memory-load tradeoff is
  (25) $$\begin{array}{c} \left(\mu ,\rho \right)=\left(1-\frac{1}{K},\;\min\left\{\frac{1}{K},\lambda \right\}\right). \end{array}$$
2. We then introduce the achievable scheme for (17) with α∈{0,1}. Assume now the K users are portioned into *g* groups of $\left\lceil \frac{K}{g}\right\rceil$ users each, where g∈[K−1]. Let the users in the same group share the same cache content and recover all the linear combinations demanded by the users in the group. Then the normalized memory-load tradeoff is as in (25) but with K replaced by with *g* and L replaced by $\left\lceil \frac{K}{g}\right\rceil L$. Therefore, we get that the following normalized memory-load points are achievable
  (26) $$\begin{array}{c} \left(\mu ,\rho \right)=\left(1-\frac{1}{g},\;\min\left\{\frac{1}{g},\lambda \left\lceil \frac{K}{g}\right\rceil \right)\right),\;g\in \left[K\right]. \end{array}$$
3. The rest of the proof of Theorem 1 consists of a method to ‘interpolate’ among the points in (26), as explained in Appendix A. Unlike cache-aided scalar linear function retrieval in [7], the difficulty in the considered problem is that connecting two normalized memory-load points by a line segment is generally impossible. The main reason is that if we partition w as $w=[w^{\prime };w^{\prime \prime }]$ and use a different cache placement strategy on each part, each demanded function $D_{k}w$ is in the form
  (27) $$\begin{array}{c} D_{k}w=D_{k}^{\prime }w^{\prime }+D_{k}^{\prime \prime }w^{\prime \prime }; \end{array}$$
  thus it cannot be divided into two separate parts, where the first part only contains the linear combinations of $w^{\prime }$ and the second part only contains the linear combinations of $w^{\prime \prime }$. An example to highlight this limitation and our approach to overcome it is provided at the end of this section.

**Remark** **5** (Comparison to the baseline scheme)**.** *We show here that the proposed scheme in Theorem 1 outperforms the baseline scheme in (3).*

- *Case $\mu =\alpha \frac{g-1}{g}+\left(1-\alpha \right)\frac{g}{g+1}$ where g∈[K] and α∈[0,1]: From (17) and (19), it can be seen that*
  (28) $$\begin{array}{c} \rho_{\mathrm{ach}}\leq \left\lceil \frac{K}{g}\right\rceil \lambda \leq K\lambda . \end{array}$$
  *From (17) and (18), it can be seen that*
  (29) $$\begin{array}{c} \rho_{\mathrm{ach}}\leq \frac{\alpha }{g}+\frac{1-\alpha }{g+1}=1-\mu . \end{array}$$
  Hence, from (28) and (29), we can prove $\rho_{\mathrm{ach}}\leq \rho_{\mathrm{baseline}}$ in this case.
- Case $\mu =\alpha \frac{K-1}{K}+\left(1-\alpha \right)$ where α∈[0,1]: Since in this case $\frac{\alpha }{K}=1-\mu$, from (20) we can prove $\rho_{\mathrm{ach}}\leq \rho_{\mathrm{baseline}}$ in this case.

**Remark** **6** (Connection to Remark 1)**.** *For the proposed scheme achieving (25), the cache placement is the same as the cache-aided scalar linear function retrieval scheme in Remark 1 with t=K−1.*

*ecalling that, in Remark 1 with t=K−1, each user misses a fraction 1/K of each file and that missing fraction is known by the remaining K−1 users; with t+1=K, the delivery consists of a single multicast message with multicasting gain K that is the sum of each user’s missing fraction of the demanded file. In our context, this idea becomes the following scheme.*

*Notice that, for the considered cache-aided general linear function retrieval problem where $\mu =\frac{t}{K}$ and t∈[K], we could use the cache-aided scalar linear function retrieval scheme in Remark 1 to deliver Kt+1 pieces of the requested vectors. The scheme would achieve*

(30) (μ,ρ)=tK,λKt+1,t∈[K],

*which reduces to (25) for t=K−1. By the grouping argument we would achieve*

(31) (μ,ρ′)=tg,λKggt+1,t∈[g],g∈[K].

*Let then fix one g∈[K] and one t∈[g−2], and analyse the achieved normalized load in (31). We will show that*

(32) ρ′=λKggt+1≥ρbaseline.

*as follows. It can be seen that*

(33) λKggt+1≥Kλgt+1g

(34) $$\begin{array}{cc} \; & \geq K\lambda \end{array}$$

(35) $$\begin{array}{cc} & \geq \rho_{\mathrm{baseline}}, \end{array}$$

*where (34) follows since t∈[g−2] and thus gt+1≥g. This shows that, with the exception for the normalized memory-load points with t=g−1, the scheme in (31) is inferior to the baseline scheme in (16), and will thus not be pursued in the rest of the paper.*

We finish this section with an example to illustrate the main ideas of the proposed scheme.

**Example** **1.** *We consider a system with* K=6 *users, cache fraction* $\mu =\frac{47}{72}$ *, and demand fraction $\lambda =\frac{1}{12}$. It can be seen that*

(36) $$\begin{array}{c} \mu =\frac{47}{72}={\left.\alpha \frac{g-1}{g}+\left(1-\alpha \right)\frac{g}{g+1}\right|}_{\alpha =\frac{1}{12},\;g=2}. \end{array}$$

*Placement Phase. It can be seen that the memory size is between* $\mu_{1}=\frac{g-1}{g}=\frac{1}{2}$ *and* $\mu_{2}=\frac{g}{g+1}=\frac{2}{3}$ *. We partition* w *into two parts as* $w=[w^{1};w^{2}]$ *where* $w^{1}\in {\left(F_{q}\right)}^{F/12}$ *and* $w^{2}\in {\left(F_{q}\right)}^{11F/12}$ *. Furthermore,*

- $w^{1}$*is partitioned into two equal-length subfiles,*$w^{1}=\left[w_{\left\{1\right\}}^{1};w_{\left\{2\right\}}^{1}\right]$*, each of which has*$\frac{F}{24}$*symbols. We divide the 6 users into 2 groups where*$G_{1}^{1}=\left\{1,3,5\right\}$*and*$G_{2}^{1}=\left\{2,4,6\right\}$*. We let users in*$G_{1}^{1}$*cache*$w_{\left\{1\right\}}^{1}$*and let users in*$G_{2}^{1}$*cache*$w_{\left\{2\right\}}^{1}$.
- $w^{2}$*is partitioned into three equal-length subfiles,*$w^{2}=\left[w_{\left\{1,2\right\}}^{2};w_{\left\{1,3\right\}}^{2};w_{\left\{2,3\right\}}^{2}\right]$*, each of which has*$\frac{11F}{36}$*symbols. We divide the 6 users into 3 groups, where*$G_{1}^{2}=\left\{1,4\right\}$, $G_{2}^{2}=\left\{2,5\right\}$, and $G_{3}^{2}=\left\{3,6\right\}$*. We let users in*$G_{1}^{2}$*cache*$w_{\left\{1,2\right\}}^{2}$*and*$w_{\left\{1,3\right\}}^{2}$, let users in $G_{2}^{2}$*cache*$w_{\left\{1,2\right\}}^{2},w_{\left\{2,3\right\}}^{2}$*, and let users in*$G_{3}^{2}$*cache*$w_{\left\{1,3\right\}}^{2}$*and*$w_{\left\{2,3\right\}}^{2}$.

*Each user caches $\frac{F}{24}+\frac{2\times 11F}{36}=\frac{47F}{72}$ symbols, thus satisfying the memory size constraint.*

*Delivery Phase. With some permutation on the rows of w, the demand of user 1 can be expressed as*

(37) $$\begin{array}{cc} D_{1}\;w= & D_{1,\{1\}}\;w_{\left\{1\right\}}^{1}+D_{1,\{1,2\}}\;w_{\left\{1,2\right\}}^{2}+D_{1,\{1,3\}}\;w_{\left\{1,3\right\}}^{2}+D_{1,\{2\}}\;w_{\left\{2\right\}}^{1}+D_{1,\{2,3\}}\;w_{\left\{2,3\right\}}^{2}. \end{array}$$

*User 1 can recover $D_{1,\{1\}}w_{\left\{1\right\}}^{1}+D_{1,\{1,2\}}w_{\left\{1,2\right\}}^{2}+D_{1,\{1,3\}}w_{\left\{1,3\right\}}^{2}$ from its cache, and similarly for the other users. Thus in the delivery phase, the users need to recover*

(38) $$\begin{array}{c} B_{1}:=D_{1,\{2\}}\;w_{\left\{2\right\}}^{1}+D_{1,\{2,3\}}\;w_{\left\{2,3\right\}}^{2}, \end{array}$$

(39) $$\begin{array}{c} B_{2}:=D_{2,\{1\}}\;w_{\left\{1\right\}}^{1}+D_{2,\{1,3\}}\;w_{\left\{1,3\right\}}^{2}, \end{array}$$

(40) $$\begin{array}{c} B_{3}:=D_{3,\{2\}}\;w_{\left\{2\right\}}^{1}+D_{3,\{1,2\}}\;w_{\left\{1,2\right\}}^{2}, \end{array}$$

(41) $$\begin{array}{c} B_{4}:=D_{4,\{1\}}\;w_{\left\{1\right\}}^{1}+D_{4,\{2,3\}}\;w_{\left\{2,3\right\}}^{2}, \end{array}$$

(42) $$\begin{array}{c} B_{5}:=D_{5,\{2\}}\;w_{\left\{2\right\}}^{1}+D_{5,\{1,3\}}\;w_{\left\{1,3\right\}}^{2}, \end{array}$$

(43) $$\begin{array}{c} B_{6}:=D_{6,\{1\}}\;w_{\left\{1\right\}}^{1}+D_{6,\{1,2\}}\;w_{\left\{1,2\right\}}^{2}. \end{array}$$

*If we treat each sum in (38)–(43) as a block and use the MAN strategy to delivery these blocks, we would transmit*$B_{1}+B_{2}$, $B_{3}+B_{4}$, $B_{5}+B_{6}$ for a total of $\frac{F}{4}$ *symbols. Hence, the scheme achieves the same normalized load as the proposed scheme in* (26) with $\mu_{1}=\frac{1}{2}$*; in other words, a portion of the memory of size* $\mu -\mu_{1}=\frac{47}{72}-\frac{1}{2}=\frac{11}{72}$ *would be wasted. We next propose two novel schemes to let each user recover its desired sum in (38)–(43) while leveraging the whole memory.*

*The solution that achieves $\rho_{1}$ in (18). Focus on the demanded sum of user 1 in (38). The key idea is to let user 1 recover $D_{1,\{2\}}w_{\left\{2\right\}}^{1}$ and $D_{1,\{2,3\}}w_{\left\{2,3\right\}}^{2}$ separately. In particular,*

- *For the first term in $B_{1}$ in (38), since the dimension of $D_{1,\{2\}}$ is $\frac{F}{12}\times \frac{F}{24}$ and the sub-demand matrix $D_{1,\{2\}}$ is known by each user, we let user 1 directly recover $w_{\left\{2\right\}}^{1}$, which contains $\frac{F}{24}$ symbols, and then compute $D_{1,\{2\}}w_{\left\{2\right\}}^{1}$. Similarly, we let users 3,5 recover $w_{\left\{2\right\}}^{1}$, and users 2,4,6 recover $w_{\left\{1\right\}}^{1}$. Thus in the delivery phase, the server transmits*
  (44) $$\begin{array}{c} w_{\left\{1\right\}}^{1}+w_{\left\{2\right\}}^{1}, \end{array}$$
  *for a total of $\frac{F}{24}$ symbols, where users 1,3,5 know $w_{\left\{1\right\}}^{1}$ and users 2,4,6 know $w_{\left\{2\right\}}^{1}$*.
- *For the second term in $B_{1}$ in (38), since the dimension of $D_{1,\{2,3\}}$ is $\frac{F}{12}\times \frac{11F}{36}$ and the sub-demand matrix $D_{1,\{2,3\}}$ is known by each user, user 1 needs to recover all symbols in $D_{1,\{2,3\}}w_{\left\{2,3\right\}}^{2}$. We denote $C_{1,\{2,3,5,6\}}^{2}:=D_{1,\{2,3\}}w_{\left\{2,3\right\}}^{2}$ since it is known by users 2,3,5,6. Hence, in order to let each user recover the first term in its desired sum, the server transmits*
  (45) $$\begin{array}{cc} \; & C_{1,\{2,3,5,6\}}^{2}+C_{2,\{1,3,4,6\}}^{2}+C_{3,\{1,2,4,5\}}^{2}, \end{array}$$
  (46) $$\begin{array}{cc} \; & C_{4,\{2,3,5,6\}}^{2}+C_{5,\{1,3,4,6\}}^{2}+C_{6,\{1,2,4,5\}}^{2}, \end{array}$$
  *for a total of $\frac{F}{6}$ symbols.*

*Hence, in the delivery phase the server transmits $\frac{F}{24}+\frac{F}{6}=\frac{5F}{24}$ symbols, and the normalized load is $\rho_{1}=\frac{5}{24}$, which coincides with (18)*.

*The solution that achieves $\rho_{2}$ in (19). The idea is to partition each user’s demand into two parts after having removed its cached content, where the partition is the result of a clever invertible linear transformation; we then have two steps, one for each of the two parts.*

*We first focus on the demand of user 1 in (38), i.e.,*

(47) $$\begin{array}{c} B_{1}=D_{1,\{2\}}w_{\left\{2\right\}}^{1}+D_{1,\{2,3\}}w_{\left\{2,3\right\}}^{2}=\left[\begin{array}{cc} D_{1,\{2\}} & D_{1,\{2,3\}} \end{array}\right]\;\left[\begin{array}{c} w_{\left\{2\right\}}^{1}\\ w_{\left\{2,3\right\}}^{2} \end{array}\right]. \end{array}$$

*The main strategy here is to take a linear transformations of (47) as follows*

(48) $$\begin{array}{c} B_{1}^{\prime }={\left(T_{1}\right)}_{\frac{F}{12}\times \frac{F}{12}}\;\left[\begin{array}{cc} {\left(D_{1,\{2\}}\right)}_{\frac{F}{12}\times \frac{F}{24}} & {\left(D_{1,\{2,3\}}\right)}_{\frac{F}{12}\times \frac{11F}{36}} \end{array}\right]\;\left[\begin{array}{c} {\left(w_{\left\{2\right\}}^{1}\right)}_{\frac{F}{24}\times 1}\\ {\left(w_{\left\{2,3\right\}}^{2}\right)}_{\frac{11F}{36}\times 1} \end{array}\right], \end{array}$$

*where $T_{1}$ is full-rank and the bottom $\frac{F}{12}-\frac{F}{24}=\frac{F}{24}$ symbols in $B_{1}^{\prime }$ are linear combinations of $w_{\left\{2,3\right\}}^{2}$ only (i.e., these linear combinations do not contain any term in $w_{\left\{2\right\}}^{1}$). This is possible because $B_{1}$ contains $\frac{F}{12}$ linear combinations of all symbols in $\left[w_{\left\{2\right\}}^{1};w_{\left\{2,3\right\}}^{2}\right]$, while $w_{\left\{2\right\}}^{1}$ contains $\frac{F}{24}$ symbols. Hence, we can re-express $B_{1}^{\prime }$ as*

(49) $$\begin{array}{c} B_{1}^{\prime }=\left[\begin{array}{c} {\left(B_{1,\{2,6\}}^{\prime }\right)}_{\frac{F}{24}\times 1}\\ {\left(B_{1,\{2,3,5,6\}}^{\prime }\right)}_{\frac{F}{24}\times 1} \end{array}\right], \end{array}$$

*where $B_{1,\{2,6\}}^{\prime }$ contains $\frac{F}{24}$ linear combinations of the symbols in $w_{\left\{2\right\}}^{1}$ and $w_{\left\{2,3\right\}}^{2}$, which are both known by users 2 and 6, while $B_{1,\{2,3,5,6\}}^{\prime }$ contains $\frac{F}{24}$ linear combinations of the symbols in $w_{\left\{2,3\right\}}^{2}$ which are known by users in {2,3,5,6}. It will be clarified later that the server transmits $B_{1,\{2,6\}}^{\prime }$ with coded caching gain equal to g=2 (i.e., the multicast message satisfies two users simultaneously), and $B_{1,\{2,3,5,6\}}^{\prime }$ with coded caching gain equal to g+1=3.*

*Following the same line or reasoning, we can express the demands of the other users as*

(50) $$\begin{array}{c} B_{2}^{\prime }=\left[B_{2,\{1,3\}}^{\prime };B_{2,\{1,3,4,6\}}^{\prime }\right]; \end{array}$$

(51) $$\begin{array}{c} B_{3}^{\prime }=\left[B_{3,\{2,4\}}^{\prime };B_{3,\{1,2,4,5\}}^{\prime }\right]; \end{array}$$

(52) $$\begin{array}{c} B_{4}^{\prime }=\left[B_{4,\{3,5\}}^{\prime };B_{4,\{2,3,5,6\}}^{\prime }\right]; \end{array}$$

(53) $$\begin{array}{c} B_{5}^{\prime }=\left[B_{5,\{4,6\}}^{\prime };B_{5,\{1,3,4,6\}}^{\prime }\right]; \end{array}$$

(54) $$\begin{array}{c} B_{6}^{\prime }=\left[B_{6,\{1,5\}}^{\prime };B_{6,\{1,2,4,5\}}^{\prime }\right]. \end{array}$$

*The transmission contains two steps.*

- *In the first step, we let each user k∈[6] recover the first term of its demand $B_{k}^{\prime }$. In this step, the server transmits*
  (55) $$\begin{array}{cc} \; & B_{1,\{2,6\}}^{\prime }+B_{2,\{1,3\}}^{\prime }, \end{array}$$
  (56) $$\begin{array}{cc} \; & B_{3,\{2,4\}}^{\prime }+B_{4,\{3,5\}}^{\prime }, \end{array}$$
  (57) $$\begin{array}{cc} \; & B_{5,\{4,6\}}^{\prime }+B_{6,\{1,5\}}^{\prime }, \end{array}$$
  *which contains $\frac{F}{8}$ symbols*.
- *In the second step, we let each user k∈[6] recover the second term of its demand $B_{k}^{\prime }$. In this step, the server transmits*
  (58) $$\begin{array}{cc} \; & B_{1,\{2,3,5,6\}}^{\prime }+B_{2,\{1,3,4,6\}}^{\prime }+B_{3,\{1,2,4,5\}}^{\prime }, \end{array}$$
  (59) $$\begin{array}{cc} \; & B_{4,\{2,3,5,6\}}^{\prime }+B_{5,\{1,3,4,6\}}^{\prime }+B_{6,\{1,2,4,5\}}^{\prime }, \end{array}$$
  *for a total of $\frac{F}{12}$ symbols. From the received multicast messages and its cache content, each user k∈[K] can recover $B_{k}^{\prime }$, and then compute $B_{k}$ from $T_{k}^{-1}B_{k}^{\prime }$*.

*The normalized load is $\rho_{2}=\frac{1}{8}+\frac{1}{12}=\frac{5}{24}$, which conincides with (19)*.

*In conclusion, the normalized load of the proposed scheme is $\rho_{\mathrm{ach}}=\min\left\{\rho_{1},\rho_{2}\right\}=\frac{5}{24}$, while the baseline scheme in (16) achieves the normalized load equals $\frac{25}{72}$.*

## 4. Numerical Evaluations

**Figure 1:**
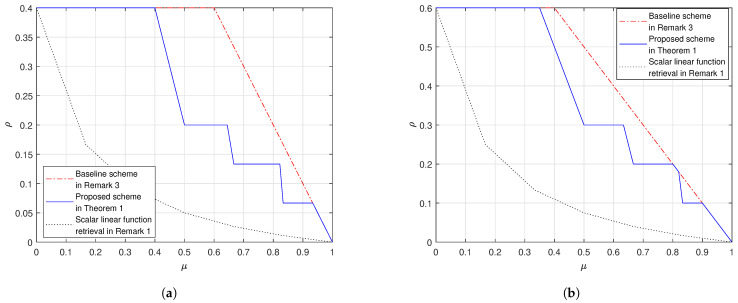
The shared-link cache-aided general linear function retrieval problem K=6. (**a**) $\lambda =\frac{1}{15}$, (**b**) $\lambda =\frac{1}{10}$.

**Figure 2:**
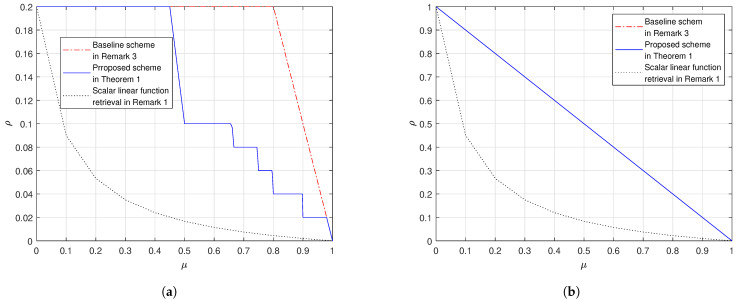
The shared-link cache-aided general linear function retrieval problem K=10. (**a**) $\lambda =\frac{1}{50}$, (**b**) $\lambda =\frac{1}{10}$.

We provide here some numerical evaluations on the performance of the proposed scheme in (17). In Figure 1a we consider the case (K,λ)=(6,1/15) and in Figure 1b the case (K,λ)=(6,1/10). In Figure 2a we consider the case (K,λ)=(10,1/50) and in Figure 2b the case (K,λ)=(10,1/10). From the figures, we observe that:

- In both settings our proposed scheme in Theorem 1 outperforms the baseline scheme, as proved in Remark 5.
- Fix K and μ. When λ grows, the gap between the proposed scheme and the baseline scheme reduces. When $\lambda =\frac{1}{K}$, the proposed scheme and the baseline scheme have the same load; this is because at the corner points of the proposed scheme in (26) we achieve the load 1−μ which is the same as the baseline scheme.
- In addition, we also plot the cache-aided scalar linear function retrieval scheme in (14), which only works for the case where the demand matrices are with the form in (12). This comparison shows that, if the demand matrices are structured, we can design caching schemes that leverage the special structure of the demands to achieve a load that is no larger than the load for the worst-case demands. Moreover, the more the structure the more the gain compared to in (17).

## 5. Conclusions

In this paper, we formulated the cache-aided general linear function retrieval problem, where each user requests some linear combinations of all the symbols in the library. The formulated problem generalizes the cache-aided scalar linear function retrieval problem. We proposed a novel scheme that strictly improves on an uncoded caching baseline scheme. Further directions include designing improved coded caching schemes for arbitrary users’ demand ranges (the setting considered here), as well as for given specific users’ demand ranges. In addition, the derivation of a converse bound is also part of on-going work.

## Appendix A. Proof of Theorem 1

By a grouping strategy, we can achieve the normalized memory-load points in (26). In the following, inspired by Example 1, we introduce a general interpolation method among the points in (26).

We let Mod(b,a) represent the modulo operation on *b* with integer divisor *a* and we let Mod(b,a)∈{1,…,a} (i.e., we let Mod(b,a)=a if *a* divides *b*).

### Appendix A.1. g∈[K−1] and $\frac{\alpha }{g}\geq \left\lceil \frac{K}{g}\right\rceil \lambda$

We first consider the case where g∈[K−1] and $\frac{\alpha }{g}\geq \left\lceil \frac{K}{g}\right\rceil \lambda$. Recall that $\mu =\alpha \frac{g-1}{g}+\left(1-\alpha \right)\frac{g}{g+1}>\frac{g-1}{g}$. In this case, we directly use the caching scheme in (26) for the memory size $\frac{g-1}{g}$ with achieved normalized load

(A1) $$\begin{array}{c} \min\left\{\left\lceil \frac{K}{g}\right\rceil \lambda ,\frac{1}{g}\right\}=\left\lceil \frac{K}{g}\right\rceil \lambda , \end{array}$$

which coincides with (17).

### Appendix A.2. g∈[K−1] and $\frac{\alpha }{g}\leq \left\lceil \frac{K}{g}\right\rceil \lambda$

We then focus on the case where g∈[K−1] and $\frac{\alpha }{g}\leq \left\lceil \frac{K}{g}\right\rceil \lambda$.

*Placement Phase.* The placement is done by the memory-sharing between the proposed placements in (26) for $M_{1}=\frac{g-1}{g}$ and $M_{2}=\frac{g}{g+1}$. We divide w into two parts, $w=[w^{1};w^{2}]$ where the dimension of $w^{1}$ is αF×1 and the dimension $w^{2}$ is (1−α)F×1.

For the first part, we further partition $w^{1}$ into *g* non-overlapping and equal-length subfiles, $w^{1}=[w_{T}^{1}:T\subseteq \left[g\right],|T|=g-1]$, where the dimension of each subfile $w_{T}^{1}$ is $\frac{\alpha F}{g}\times 1$. Each user k∈[K] caches $w_{T}^{1}$ where T⊆[g], |T|=g−1, and Mod(k,g)∈T.

For the second part of each file, we further partition $w^{2}$ into g+1 non-overlapping and equal-length subfiles, $w^{2}=[w_{T}^{2}:T\subseteq \left[G+1\right],|T|=g]$, where the dimension of each subfile $w_{T}^{2}$ is $\frac{(1-\alpha )F}{g+1}\times 1$. Each user k∈[K] caches $w_{T}^{2}$ where T⊆[g+1], |T|=G, and Mod(k,g+1)∈T.

In total, each user caches

(A2) $$\begin{array}{c} \left(g-1\right)\frac{\alpha F}{g}+\frac{g(1-\alpha )F}{g+1}=\mu F \end{array}$$

symbols, satisfying the memory size constraint.

*Delivery Phase.* For each $T_{1}\in \left[g\right]$ where $|T_{1}|=g-1$, we define $D_{k,T_{1}}$ as the sub-matrix of $D_{k}$ which contains the columns corresponding to the symbols in $w_{T_{1}}^{1}$. In addition, for each $T_{2}\in \left[g+1\right]$ where $|T_{2}|=g$, we define $D_{k,T_{2}}$ as the sub-matrix of $D_{k}$ which contains the columns corresponding to the symbols in $w_{T_{2}}^{2}$.

We can express the demand of user k∈[K] as

(A3) $$\begin{array}{cc} D_{k}\;w & =\sum_{T_{1}\in \left[g\right]:\left|T_{1}\right|=g-1}D_{k,T_{1}}w_{T_{1}}^{1}+\sum_{T_{2}\in \left[g+1\right]:\left|T_{2}\right|=g}D_{k,T_{2}}w_{T_{2}}^{2}\\ \; & =\sum_{\begin{array}{c} T_{1}\in \left[g\right]:\left|T_{1}\right|=g-1,\\ \mathrm{Mod}\left(k,g\right)\in T_{1} \end{array}}D_{k,T_{1}}w_{T_{1}}^{1}+\sum_{\begin{array}{c} T_{2}\in \left[g+1\right]:\left|T_{2}\right|=g,\\ \mathrm{Mod}\left(k,g+1\right)\in T_{2} \end{array}}D_{k,T_{2}}w_{T_{2}}^{2} \end{array}$$

(A4) $$\begin{array}{cc} \; & +D_{k,[g]\backslash \{\mathrm{Mod}(k,g)\}}\;w_{\left[g]\backslash \{\mathrm{Mod}(k,g)\right\}}^{1}+D_{k,[g+1]\backslash \{\mathrm{Mod}(k,g+1)\}}\;w_{\left[g+1]\backslash \{\mathrm{Mod}(k,g+1)\right\}}^{2}. \end{array}$$

It can be seen that user *k* knows all the terms in (A4) except

(A5) $$\begin{array}{c} B_{k}:=D_{k,[g]\backslash \{\mathrm{Mod}(k,g)\}}\;w_{\left[g]\backslash \{\mathrm{Mod}(k,g)\right\}}^{1}+D_{k,[g+1]\backslash \{\mathrm{Mod}(k,g+1)\}}\;w_{\left[g+1]\backslash \{\mathrm{Mod}(k,g+1)\right\}}^{2}. \end{array}$$

Hence, in the delivery phase user *k* should recover $B_{k}$. We then propose two solutions for this objective.

*The solution that achieves $\rho_{1}$ in* (18). We let user *k* recover

(A6) $$\begin{array}{cc} \; & B_{k,1}:=D_{k,[g]\backslash \{\mathrm{Mod}(k,g)\}}\;w_{\left[g]\backslash \{\mathrm{Mod}(k,g)\right\}}^{1}, \end{array}$$

(A7) $$\begin{array}{cc} \; & B_{k,2}:=D_{k,[g+1]\backslash \{\mathrm{Mod}(k,g+1)\}}\;w_{\left[g+1]\backslash \{\mathrm{Mod}(k,g+1)\right\}}^{2}. \end{array}$$

For the first term $B_{k,1}$, the dimension of $D_{k,[g]\backslash \{\mathrm{Mod}(k,g)\}}$ is $\lambda F\times \frac{\alpha F}{g}$ and $D_{k,[g]\backslash \{\mathrm{Mod}(k,g)\}}$ is known by each user. Recall that in this case we have $\frac{\alpha F}{g}\leq \lambda F$. Hence, we let user *k* directly recover $w_{\left[g]\backslash \{\mathrm{Mod}(k,g)\right\}}^{1}$. Thus in the delivery phase, we let the server transmit

(A8) $$\begin{array}{c} \sum_{i\in [g]}w_{\left[g]\backslash \{i\right\}}^{1}, \end{array}$$

with $\frac{\alpha F}{g}$ symbols. It can be seen that each user k∈[K] desires $w_{\left[g]\backslash \{\mathrm{Mod}(k,g)\right\}}^{1}$ and caches all the other terms in (A8), such that user *k* can recover $w_{\left[g]\backslash \{\mathrm{Mod}(k,g)\right\}}^{1}$.

For the second term $B_{k,2}$, the dimension of $D_{k,[g+1]\backslash \{\mathrm{Mod}(k,g+1)\}}$ is $\lambda F\times \frac{(1-\alpha )F}{g+1}$. Notice that $B_{k,2}$ only contains linear combinations of the second parts of files in the library. For the second part of each file, the users in

$$G_{i}^{2}=\left\{k\in \left[K\right]:\mathrm{Mod}\left(k,g+1\right)=i\right\}$$

cache the same content, where i∈[g+1]. Thus we can use the proposed delivery scheme in (26). More precisely, for each i∈[g+1], we generate a virtual user $v_{i}$ with the demand

(A9) $$\begin{array}{c} D_{i}^{\prime }\;w_{\left[g+1]\backslash \{i\right\}}^{2}=\left[\begin{array}{c} D_{G_{i}^{2}\left(1\right),\left[g+1\right]\backslash \left\{i\right\}}\\ D_{G_{i}^{2}\left(2\right),\left[g+1\right]\backslash \left\{i\right\}}\\ \vdots \\ D_{G_{i}^{2}\left(\right|G_{i}^{2}\left|\right),\left[g+1\right]\backslash \left\{i\right\}} \end{array}\right]\;w_{\left[g+1]\backslash \{i\right\}}^{2}. \end{array}$$

Notice that the dimension of $D_{i}^{\prime }$ is $|G_{i}^{2}|\lambda F\times \frac{(1-\alpha )F}{g+1}$. So virtual user $v_{i}$ only needs to recover at most $\min\left\{\left\lceil \frac{K}{g+1}\right\rceil \lambda ,\frac{1-\alpha }{g+1}\right\}F$ symbols in (A9). We denote the set of these symbols by $P_{i,[g+1]\backslash \{i\}}^{\prime }$, which is known by all the other virtual users. We then let the server transmit

(A10) $$\begin{array}{c} \sum_{i\in [g+1]}P_{i,[g+1]\backslash \{i\}}^{\prime }, \end{array}$$

with $\min\left\{\left\lceil \frac{K}{g+1}\right\rceil \lambda ,\frac{1-\alpha }{g+1}\right\}F$ symbols, such that each virtual user can recover its demand.

In total, the server transmits

(A11) $$\begin{array}{c} \frac{\alpha F}{g}+\min\left\{\left\lceil \frac{K}{g+1}\right\rceil \lambda ,\frac{1-\alpha }{g+1}\right\}F=\rho_{1}F \end{array}$$

symbols, which coincides with (18).

*The solution that achieves $\rho_{2}$ in (19).* Recall that the demanded sum of user *k* is

(A12) $$\begin{array}{cc} B_{k} & =D_{k,[g]\backslash \{\mathrm{Mod}(k,g)\}}\;w_{\left[g]\backslash \{\mathrm{Mod}(k,g)\right\}}^{1}+D_{k,[g+1]\backslash \{\mathrm{Mod}(k,g+1)\}}\;w_{\left[g+1]\backslash \{\mathrm{Mod}(k,g+1)\right\}}^{2} \end{array}$$

(A13) $$\begin{array}{cc} \; & =\left[\begin{array}{cc} D_{k,[g]\backslash \{\mathrm{Mod}(k,g)\}} & \;D_{k,[g+1]\backslash \{\mathrm{Mod}(k,g+1)\}} \end{array}\right]\;\left[\begin{array}{c} w_{\left[g]\backslash \{\mathrm{Mod}(k,g)\right\}}^{1}\\ w_{\left[g+1]\backslash \{\mathrm{Mod}(k,g+1)\right\}}^{2} \end{array}\right]. \end{array}$$

We can take a linear transformations on $B_{k}$ as follows

(A14) $$\begin{array}{c} B_{k}^{\prime }=T_{k}\;\left[\begin{array}{cc} D_{k,[g]\backslash \{\mathrm{Mod}(k,g)\}} & \;D_{k,[g+1]\backslash \{\mathrm{Mod}(k,g+1)\}} \end{array}\right]\;\left[\begin{array}{c} w_{\left[g]\backslash \{\mathrm{Mod}(k,g)\right\}}^{1}\\ w_{\left[g+1]\backslash \{\mathrm{Mod}(k,g+1)\right\}}^{2} \end{array}\right], \end{array}$$

where $T_{k}$ is full-rank with dimension λF×λF, and the bottom ${\left[\lambda -\frac{\alpha }{g}\right]}^{+}F$ symbols in $B_{k}^{\prime }$ are some linear combinations of $w_{\left[g+1]\backslash \{\mathrm{Mod}(k,g+1)\right\}}^{2}$ (i.e., these linear combinations do not contain any term in $w_{\left[g]\backslash \{\mathrm{Mod}(k,g)\right\}}^{1}$). This is possible because $B_{k}$ contains λF linear combinations of all symbols in $\left[w_{\left[g]\backslash \{\mathrm{Mod}(k,g)\right\}}^{1};w_{\left[g+1]\backslash \{\mathrm{Mod}(k,g+1)\right\}}^{2}\right]$, while $w_{\left[g]\backslash \{\mathrm{Mod}(k,g)\right\}}^{1}$ contains $\frac{\alpha F}{g}$ symbols. Hence, we can re-express $B_{k}^{\prime }$ as

(A15) $$\begin{array}{c} B_{k}^{\prime }=\left[\begin{array}{c} {\left(B_{k,1}^{\prime }\right)}_{\min\left\{\frac{\alpha F}{g},\lambda F\right\}\times 1}\\ {\left(B_{k,2}^{\prime }\right)}_{{\left[\lambda -\frac{\alpha }{g}\right]}^{+}F\times 1} \end{array}\right]. \end{array}$$

The delivery phase is divided into two steps. In the first step, we first let each user k∈[K] recover $B_{k,1}^{\prime }$. Notice that $B_{k,1}^{\prime }$ is the set of some linear combinations of the symbols in $w_{\left[g]\backslash \{\mathrm{Mod}(k,g)\right\}}^{1}w_{\left[g+1]\backslash \{\mathrm{Mod}(k,g+1)\right\}}^{2}$. $w_{\left[g]\backslash \{\mathrm{Mod}(k,g)\right\}}^{1}$ is known by any user $j_{1}\in \left[K\right]$ where $\mathrm{Mod}(j_{1},g)\neq k$; $w_{\left[g+1]\backslash \{\mathrm{Mod}(k,g+1)\right\}}^{2}$ is known by any user $j_{2}\in \left[K\right]$ where $\mathrm{Mod}(j_{2},g+1)\neq k$. Assume that $k=a_{k}g+\mathrm{Mod}\left(k,g\right)$, where $a_{k}=\left\lceil \frac{k}{g}\right\rceil -1$ and Mod(k,g)∈[g]. In Appendix B, we prove the following lemma.

**Lemma** **A1.**
*Each user $k_{1}=a_{k}g+j$ where j∈[g]\{Mod(k,g)} and $k_{1}\in \left[K\right]$, caches both $w_{\left[g]\backslash \{\mathrm{Mod}(k,g)\right\}}^{1}$ and $w_{\left[g+1]\backslash \{\mathrm{Mod}(k,g+1)\right\}}^{2}$.*

For each $i\in \left[\left\lceil \frac{K}{g}\right\rceil \right]$, we let the server transmit

(A16) $$\begin{array}{c} \sum_{j\in [g]:(i-1)g+j\leq K}B_{(i-1)g+j,1}^{\prime }. \end{array}$$

From Lemma A1, each user (i−1)g+j knows all except $B_{(i-1)g+j,1}^{\prime }$ such that it can recover $B_{(i-1)g+j,1}^{\prime }$. In this step, the server transmits $\left\lceil \frac{K}{g}\right\rceil \min\left\{\frac{\alpha }{g},\lambda \right\}F$ symbols.

In the second step, we then let each user k∈[K] recover $B_{k,2}^{\prime }$, which contains linear combinations of $w_{\left[g+1]\backslash \{\mathrm{Mod}(k,g+1)\right\}}^{2}$. We can use the same delivery scheme as we used to delivery the second term in the first solution (i.e., $B_{k,2}$ in (A7) which contains λF linear combinations of $w_{\left[g+1]\backslash \{\mathrm{Mod}(k,g+1)\right\}}^{2}$). Here we do not repeat the scheme. Notice that $B_{k,2}^{\prime }$ contains ${\left[\lambda -\frac{\alpha }{g}\right]}^{+}F$ linear combinations of $w_{\left[g+1]\backslash \{\mathrm{Mod}(k,g+1)\right\}}^{2}$. Hence, in this step the server transmits $\min\left\{\left\lceil \frac{K}{g+1}\right\rceil {\left[\lambda -\frac{\alpha }{g}\right]}^{+},\frac{1-\alpha }{g+1}\right\}F$ symbols.

After recovering $B_{k}^{\prime }$, each user k∈[K] reconstructs

$$B_{k}=T_{k}^{-1}B_{k}^{\prime },$$

and then recovers its demand.

In total, the achieved normalized load is

$$\begin{array}{c} \left\lceil \frac{K}{g}\right\rceil \min\left\{\frac{\alpha }{g},\lambda \right\}+\min\left\{\left\lceil \frac{K}{g+1}\right\rceil {\left[\lambda -\frac{\alpha }{g}\right]}^{+},\frac{1-\alpha }{g+1}\right\}=\rho_{2}, \end{array}$$

coinciding with (19).

### Appendix A.3. Proof of (20)

Finally, we focus on the case $\mu =\alpha \frac{K-1}{K}+\left(1-\alpha \right)$ where α∈(0,1). In this case, the proposed scheme is a direct extension from the proposed scheme in (25). More precisely,

- we directly use the caching scheme in (25) for the memory size $\frac{K-1}{K}$ with the achieved normalized load equal to λ.
- In this case, the number of symbols which are not cached by user is $\frac{\alpha F}{K}$. Hence, we can let each user directly recover the uncached symbols with the achieved normalized load equal to $\frac{\alpha }{K}$.

This concludes the proof.

## Appendix B. Proof of Lemma A1

Recall that $k=a_{k}g+\mathrm{Mod}\left(k,g\right)$, where $a_{k}=\left\lceil \frac{k}{g}\right\rceil -1$ and Mod(k,g)∈[g]. We focus on one user $k_{1}=a_{k}g+j$ where j∈[g]\{Mod(k,g)}. Since $j=\mathrm{Mod}\left(k_{1},g\right)\neq \mathrm{Mod}\left(k,g\right)$, it can be easily seen that $w_{\left[g]\backslash \{\mathrm{Mod}(k,g)\right\}}^{1}$ is cached by user *j*. In the rest of this proof, we show that user *j* also caches $w_{\left[g+1]\backslash \{\mathrm{Mod}(k,g+1)\right\}}^{2}$; or equivalently, $\mathrm{Mod}\left(k_{1},g+1\right)\neq \mathrm{Mod}\left(k,g+1\right)$.

We prove it by contradiction. Assume that $\mathrm{Mod}\left(k_{1},g+1\right)=\mathrm{Mod}\left(k,g+1\right)=j^{\prime }$. Hence, we can re-express *k* as $k=a_{k}^{\prime }\left(g+1\right)+j^{\prime }$ and re-express $k_{1}$ as $k_{1}=a_{k_{1}}^{\prime }\left(g+1\right)+j^{\prime }$, where $a_{k}^{\prime }=\left\lceil \frac{k}{g+1}\right\rceil -1$ and $a_{k_{1}}^{\prime }=a_{k}^{\prime }=\left\lceil \frac{k_{1}}{g+1}\right\rceil -1$.

Since $k=a_{k}g+\mathrm{Mod}\left(k,g\right)=a_{k}^{\prime }\left(g+1\right)+j^{\prime }$, we have

(A17) $$\begin{array}{c} a_{k}g=a_{k}^{\prime }\left(g+1\right)+j^{\prime }-\mathrm{Mod}\left(k,g\right). \end{array}$$

In addition, we have

(A18) $$\begin{array}{c} k_{1}=a_{k}g+j=a_{k_{1}}^{\prime }\left(g+1\right)+j^{\prime } \end{array}$$

By taking (A17) into (A18), we have

(A19) $$\begin{array}{cc} \; & a_{k}g+j=a_{k_{1}}^{\prime }\left(g+1\right)+j^{\prime } \end{array}$$

(A20) $$\begin{array}{cc} \; & \overset{\left(\right)}{⟺}a_{k}^{\prime }\left(g+1\right)+j^{\prime }-\mathrm{Mod}\left(k,g\right)+j=a_{k_{1}}^{\prime }\left(g+1\right)+j^{\prime } \end{array}$$

(A21) $$\begin{array}{cc} \; & ⟺\left(a_{k}^{\prime }-a_{k_{1}}^{\prime }\right)\left(g+1\right)=\mathrm{Mod}\left(k,g\right)-j. \end{array}$$

Since Mod(k,g)∈[g] and j∈[g], it can be seen that (A19) holds if and only if $a_{k}^{\prime }-a_{k_{1}}^{\prime }=0$, which leads to $k=k_{1}$ and contradicts with $\mathrm{Mod}\left(k,g\right)\neq \mathrm{Mod}\left(k_{1},g\right)$. Hence, we proved that $\mathrm{Mod}\left(k_{1},g+1\right)\neq \mathrm{Mod}\left(k,g+1\right)$ and proved Lemma A1.
